# Essential health services delivery and quality improvement actions under drought and food insecurity emergency in north-east Uganda

**DOI:** 10.1186/s12913-023-10377-y

**Published:** 2023-12-11

**Authors:** Charles Njuguna, Habteyes Hailu Tola, Benson Ngugi Maina, Kwikiriza Nicholas Magambo, Nabunya Phoebe, Evelyne Tibananuka, Florence M. Turyashemererwa, Moses Rubangakene, Kisubika Richard, George Opong, Ssekitoleko Richard, Chris Opesen, Tim Mateeba, Edmond Muyingo, Upenytho George, Samalie Namukose, Yonas Tegegn Woldemariam

**Affiliations:** 1 World Health Organization Uganda Country Office, Plot 60 Prince Charles Drive, Kololo, P.O. Box 24578, Kampala, Uganda; 2 Ministry of Health of Uganda, Kampala, Uganda

**Keywords:** Health system, Health services, Quality improvement

## Abstract

**Background:**

Essential health services can be disrupted due to several naturally occurring public health emergencies such as drought, flood, earthquake and outbreak of infectious diseases. However, little evidence exists on the status of essential health services delivery under the effect of drought and food insecurity. North-east Uganda is severely affected by prolonged drought that significantly affected the livelihood of the residents. Therefore, we aimed to determine the current status of essential health services and quality improvement (QI) actions in health facilities in north-east Uganda.

**Methods:**

We used a descriptive cross-sectional study design to assess the availability of essential health service and quality improvement activities in drought and food insecurity affected districts of north-east Uganda. We included a total of 150 health facilities from 15 districts with proportionated multistage sampling method. We interviewed health facilities’ managers and services focal persons using structured questionnaire and observation checklist. We used a descriptive statistic to analyze the data with SPSS version 22.

**Results:**

A few health facilities (8.7%) had mental health specialist. There was also lack of capacity building training on essential health services. Considerable proportion of health facilities had no non-communicable diseases (38.3%), mental health (47.0%), and basic emergency obstetric care (40.3%) services. Stock out of essential medicines were observed in 20% of health facilities. There was lack of supportive supervision, and poor documentation of QI activities.

**Conclusion:**

Essential health service and QI were suboptimal in drought and food insecure emergency affected districts. Human resource deployment (especially mental health specialist), provision of capacity building training, improving non-communicable diseases, mental health and basic emergency obstetric care services are required to improve availability of essential health services. Supporting supply chain management to minimize stock out of medicines, and promoting QI activities are also vital to assure quality of health service in drought and food insecurity affected districts in north-Eastern Uganda.

## Background

A properly functioning healthcare system is a fundamental requirement to deliver quality health services based on the demand of the clients. It is also important to achieve a universal coverage of healthcare which recently became a focal point of advocacy across the world, due to its importance in achieving health related sustainable development goal targets [1, 2]. A properly functioning healthcare system encompasses the availability of physical infrastructure, skilled human power and financial resources to deliver healthcare services that fulfill the demands of a population under that catchment area. However, healthcare system is offering suboptimal services due to several factors such as lack of physical infrastructure [3], absence of essential healthcare services in the facility [4], lack of skilled human power [5] and lack of financial resource [6], poor governance and supply stockout [7]. Furthermore, healthcare system can be disrupted by the occurrence of natural disasters and disease outbreaks. For instance, the effect of COVID-19 pandemic on the global health system is an ongoing public health problem which significantly affected health service delivery systems across the world [8–10]. Several healthcare facilities have been shifted to COVID-19 pandemic response, and essential healthcare service delivery was disrupted due to human and financial resources being diverted to respond to the pandemic [11]. Health system in low-and-middle-income countries (LMICs) was specifically affected by COVID-19 pandemic due to poor resilience to the impact of the pandemic [12, 13].

The occurrence of man-made and natural disasters such as drought, flood, earthquake and conflict also have a considerable effect on health system. For example, drought imposes food insecurity on household, which leads to malnutrition and deaths of several children in different setups [14, 15]. It also leads to an upsurge of diarrheal and vector borne diseases [16–18] due to water shortage for hygiene, and through creating conducive environment for infectious agents breeding. The upsurge of malnutrition, diarrheal and vector borne disease could overburden the healthcare facilities and affect the usual health service delivery process. The frequency of the occurrence of communicable diseases outbreaks could also increase during the drought season [16–18] which could lead to high burden of case load in the healthcare system and disrupt the essential health services delivery. For these reasons, regular health service delivery system status assessment is important for planning efficient intervention under drought and food insecurity emergency.

Drought and food insecurity emergency pose challenges to essential health services delivery system across the world [18, 19]. Its effect is more pronounced in low income countries where the health system is not efficient. The Great Horn of Africa (GHoA) is one of the regions that is persistently affected by food insecurity emergencies due to drought, floods, and conflicts [20]. Millions of people in GHoA are facing severe food insecurity and infectious diseases outbreaks [21], with thirty seven million people facing acute hunger and nearly seven million children under the age of five year suffering from acute malnutrition [22]. Beside malnutrition, the occurrence of cholera, measles, malaria and yellow fever have complicated the responses to drought and food insecurity crisis in the GHoA region and severely impacted health service delivery [20].

Uganda is one of the countries in GHoA region with its north-eastern part being affected by drought and food insecurity [20]. Nine districts in Karamoja region are categorized under phase 3 or higher food insecurity emergency, and 42% of population in the area are suffering from acute food insecurity [23]. Although the government with development partners are responding to the public health consequences of drought and food insecurity, essential health services delivery systems in this region is still being affected. There is also evidence gap on the status of essential health services under the impact of drought and food insecurity emergeny in this region to support the response efforts. Therefore, we aimed to assess the current status of essential health services and health service quality improvement actions in drought and food insecurity affected districts in north-east Uganda.

## Methods

### Study design and area

We employed a descriptive cross-sectional study design to determine the current status of essential health services and service quality improvement actions in Karamoja region and surrounding districts in north-east Uganda. Descriptive cross-sectional study design is an appropriate study design to achieve the aim of this study. Karamoja region is severely affected by drought and food insecurity which classified at integrated food security phase classification (IPC) phase 3 and above food insecurity conditions [24]. The drought and food insecurity emergency also affected districts surrounding Karamoja region to some extent, although the effect is not the same as in Karamoja region.

**Fig. 1:**
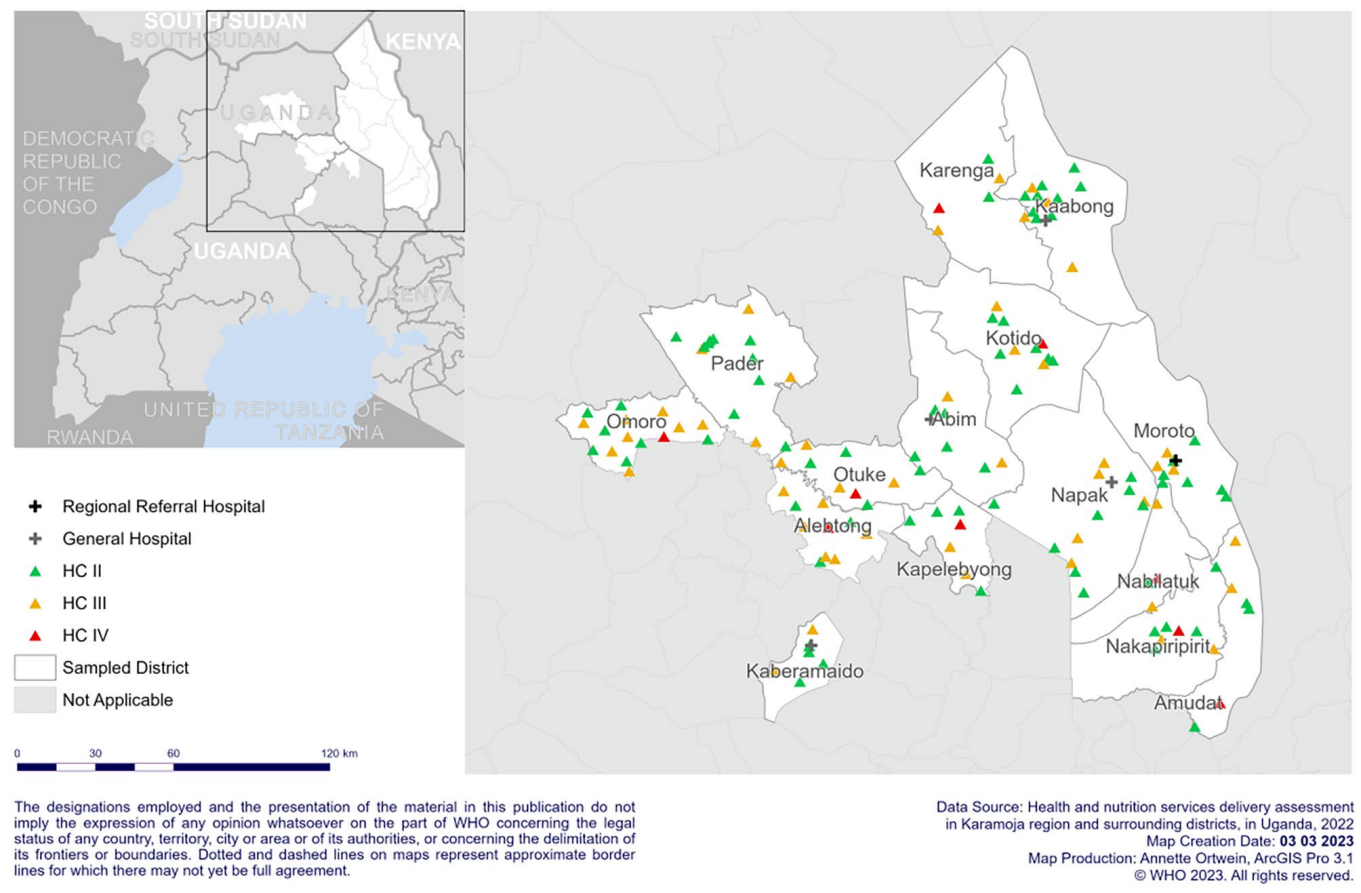
Study area and distribution of selected health facilities in Karamoja region and surrounding districts

The effect of drought and food insecurity in the region significantly affected essential health services delivery process and quality of care. The region is also reporting high burden of infectious diseases and malnutrition cases which have significantly impacted the health service delivery and quality of care. Provision of quality essential health services could be also affected by staff-turnover and insecurity due to warriors and riders in the region as the result of drought. Thus, we conducted this study in 15 selected districts that are affected by drought and food insecurity [Fig. 1].

### Sample size calculation and sampling method

**Fig. 2:**
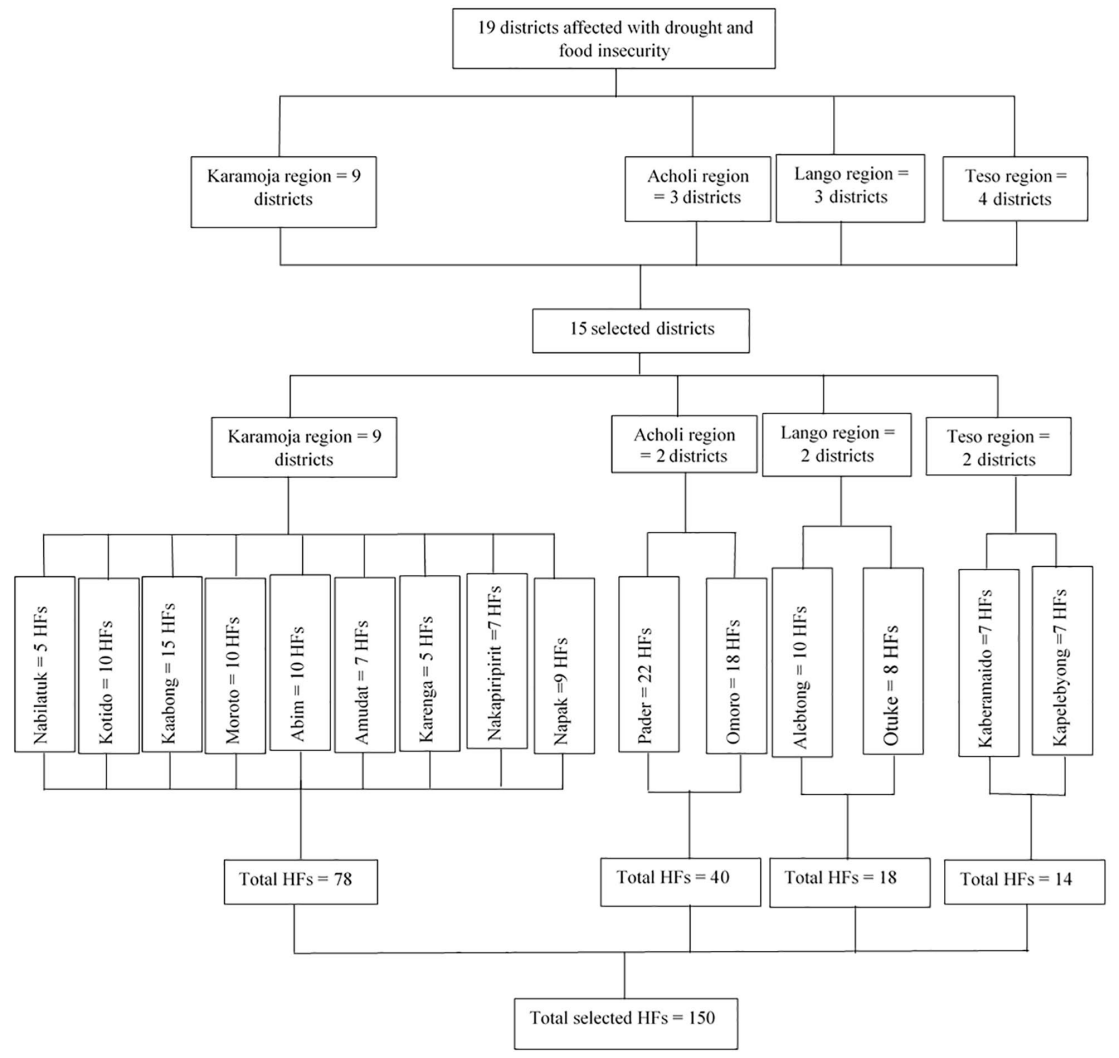
Sample size and sampling procedure of the districts and health facilities (HFs-health facilities)

Figure 2 depicts sample size calculation and sampling procedure of districts and health facilities. We used multistage proportionated sampling technique to select the districts and health facilities [Fig. 1]. The first sampling stage was the four drought affected regions (Acholi, Karamoja, Lango and Teso), while the second to be sampled were districts in the regions, and the third being health facilities in the districts. In the first stage we included all the four regions that were affected by drought and food insecurity using purposive sampling method. In the second stage, we included 50% of districts from three regions (Acholi, Lango and Teso) by simple random sampling [Fig. 2]. However, we included all nine districts of Karamoja region, because all districts in the region were severely affected by drought and food insecurity [Fig. 2]. This could help the policy makers and intervention implementing partners to understand the current status of essential health services under the effect of drought and food insecurity condition. We included a total of 15 (78.9%) districts in this study from 19 districts affected by drought and food insecurity emergency in north-east Uganda [Fig. 2]. We finally included 150 (53.4%) health facilities [Figs. 1 and 2] in the study out of 281 total health facilities located in the selected 15 districts using simple random sampling method [Fig. 2]. We selected more than 50% of the health facility within health center level II and III by simple random sampling with replacement for inactive health centers (HCs) because health centers level II and III are more in number. However, we included nine HCs level IV and seven hospitals in the selected districts purposely, because the selected districts have few HCs level IV and hospitals.

### Inclusion and exclusion criteria

We included all active public health facilities in the selected districts and excluded private health facilities because they try to provide health service under any circumstance compare to public facilities.

### Data collection tools and methods

We used structured questionnaires and observation checklists adopted from the Ugandan nutrition services quality assessment [25] and World Health Organization (WHO) harmonized health facility assessment tools [26]. All questions were checked for clarity and relevancy by experienced public health professionals at WHO, Ministry of Health (MoH) and district health offices. In addition, the questionnaires were piloted in two health facilities that were different from the main assessment health facilities, and the problems identified in the questionnaires were corrected before commencement of data collection.

We interviewed health facility managers and service focal persons of each health facility to collect necessary information on each service. Health facility managers or service focal persons were also guided the observation of the services and the facilities in the health facilities. Data was collected by health professionals from district health offices and MoH after they obtained two days training on the objectives of the study and data collection tools. The district health officers (DHOs) were assigned in different districts from where they were working during the study period to minimize interviewer bias. All data was entered into open data kit (ODK) and checked daily for errors and in the event of errors, corrective measures were taken by data collection supervisors at the field and data manager at WHO country office through remote access.

### Data analysis

We used descriptive statistics such as frequency, percentage, mean with standard deviation, and median with interquartile range to summarize the data. To show estimation precision, we estimated 95% confidence interval around the proportions. We used statistical package for social scientists (SPSS) version 22 to analyze the data.

## Results

### Interviewees and health facilities characteristics

Of 150 included health facilities, 78 (52%) were level II health centers (HC-II), 56 (37.3%) level III (HC-III), 9 (6.0%) level IV (HC-IV) and 7 (4.7%) hospitals. Of the interviewed professions, 82 (54.7%) were nurses, 35 (23.3%) midwives, 26 (17.3%) clinical officers, 4 (2.7%) medical officers, and 3 (2.0%) laboratory personnel.

### Human resource availability

Table 1 depicts the human resource capacity of the assessed health facilities. Of the 150 health facilities, 135 (90%) had nurses, and 127 (84.7%) had midwives. Seventy-two (48%) of health facilities had health information assistants and only 13 (8.7%) had mental health specialists. Moreover, 80 (53.3%) of health facilities had no laboratory personnel and all level IV HCs and hospitals had at least one medical officer, however, none of level II and III had medical officers. In general, most of the healthcare workers were concentrated in HCs level IV and hospitals [Table 1].

**Table 1:** Availability of health professionals across the levels of health facilities

Profession		Level of HF, n (%)				Total (n* = 150)
		HC-II (n = 78)	HC-III (n = 56)	HC-IV (n = 9)	Hospital (n = 7)	
Nurses	Yes	69 (88.5)	50 (89.3)	9 (100)	7 (100)	135(90.0)
	No	9 (60.0)	6 (40.0)	0 (0.0)	0 (0.0)	15 (10.0)
Midwive	Yes	61(48.0)	50 (39.4)	9 (7.1)	7 (5.5)	127 (84.7)
	No	17 (73.9)	6 (26.1)	0 (0.0)	0 (0.0)	23(15.3)
Health information assistants	Yes	12 (16.7)	44 (61.1)	9 (12.2)	7 (9.7)	72 (48.0)
	No	66 (84.6)	12 (15.4)	0 (0.0)	0 (0.0)	78 (52.0)
Nursing assistants	Yes	43 (44.3)	39 (40.2)	8 (8.2)	7 (7.2)	97 (64.7)
	No	35 (66.0)	17 (32.1)	1 (1.9)	0 (0.0)	53 (35.3)
Medical officers (n = 16)	Yes	----	----	9 (45.0)	7(35.0)	16 (100)
	No	----	-----	0 (0.0)	0 (0.0)	0 (0.0)
Laboratory personnel	Yes	10 (14.3)	44 (62.9)	9 (12.9)	7 (10.0)	70 (46.7)
	No	68 (85.0)	12 (15.0)	0 (0.0)	0 (0.0)	80 (53.3)
Pharmacist/dispensers	Yes	3 (16.7)	4 (22.2)	5 (27.8)	6 (33.3)	18 (12.0)
	No	75 (56.8)	52 (39.4)	4 (3.0)	1(0.8)	132 (88.0)
Mental health specialists	Yes	1(7.7)	0 (0.0)	7 (53.8)	5 (38.5)	13 (8.7)
	No	77 (56.2)	56 (40.9)	2 (1.5)	2 (1.5)	137 (91.3)
Anesthesiologists (n = 16)	Yes	-----	----	6 (37.5)	7 (43.8)	13 (81.3)
	No	------	----	3 (18.8)	0 (0.0)	3 (18.7)
Radiologists (n = 16)	Yes	-----	-----	2 (12.5)	2 (12.5)	4 (25.0)
	No	-----	------	7 (43.8)	5 (31.3)	12 (75.0)

*-denominator is 150, unless indicated; HC-health center

### Capacity building of healthcare workers

#### Training on basic emergency obstetric care

Of the 150 assessed health facilities, 79 (52.7%) had someone who trained on basic emergency obstetric care (BEmOC) in past two years [Table 2]. Of the 79 health facilities which had someone who trained on BEmOC, majority (96.2%) had midwives [Table 2]. Of the 16 health facilities that were supposed to have medical officers based on the national standard, only 5 (31.3%) had medical officers who trained on BEmOC in past two years [Table 2]. A small proportion of health facilities had nursing assistants (2.5%), and clinical officer (5.1%) who trained on BEmOC in past two years [Table 2].

**Table 2:** Basic emergency obstetric care training distribution in health facilities (n = 79)

Profession		Frequency	% (95% CI)
Nurses	Trained	9	11.4 (5.9–20.5)
	Not trained	70	88.6 (79.5–94.1)
Midwives	Trained	76	96.2 (89.0–99.2)
	Not trained	3	3.8 (0.9–11.0)
Nursing assistants	Trained	2	2.5 (0.2–9.3)
	Not trained	77	97.5 (90.7–99.4)
Clinical officers	Trained	4	5.1 (1.6–12.7)
	Not trained	75	94.9 (87.3–98.4)
Medical officers (n = 16)	Trained	5	31.3 (13.9–55.9)
	Not trained	11	68.7 (44.2–86.1)

*CI-Confidence interval*

#### Training on human immunodeficiency virus (HIV) and Tuberculosis (TB)

Table 3 depicts the distribution of training obtained in past two years on HIV and TB in the included 150 health facilities. Of the total 150 assessed health facilities, 88 (58.7%) had someone who trained on HIV diagnosis and care in past two years. Majority (76.1%) of health facilities had nurses, midwives (54.5%) and medical officers (50.0%) who trained on HIV diagnosis and care in past two years. Of the 88-health facility which had someone trained on HIV| diagnosis and treatment, only 20 (22.7%) of the health facilities had laboratory personnel, pharmacists 2 (2.3%), and nursing assistants 11 (112.5%) who trained on HIV diagnosis and treatment [Table 3].

**Table 3:** Distribution of training on HIV and tuberculosis care in past two years in health facilities

Profession		HIV (n = 88)		Tuberculosis (n = 98)	
		Frequency	% (95% CI)	Frequency	% (95% CI)
Nurses	Trained	67	76.1 (66.2–83.9)	72	73.5 (63.9–81.3)
	Not trained	21	23.9 (16.1–33.8)	26	26.5 (18.8–36.1)
Midwives	Trained	48	54.5 (44.2–64.5)	29	29.6 (21.4–39.3)
	Not trained	40	45.5 (35.5–55.8)	69	70.4 (60.7–78.6)
Nursing assistants	Trained	11	12.5 (7.0–21.2)	9	9.2 (4.7–16.7)
	Not trained	77	87.5 (78.8–93.0)	89	90.8 (83.2–95.3)
Clinical officers	Trained	31	35.2 (26.0–45.7)	37	37.8 (28.8–47.7)
	Not trained	57	64.8 (54.4–74.0)	61	62.2 (52.4–71.2)
Medical officers (n = 16)	Trained	8	50.0 (28.0–72.0)	9	56.3 (33.2–76.9)
	Not trained	8	50.0 (28.0–72.0)	7	43.8 (23.1–66.9)
Laboratory personnel	Trained	20	22.7 (15.2–32.6)	32	32.7 (24.2–42.5)
	Not trained	68	77.3 (67.4–84.9)	66	67.3 (57.5–75.9)
Pharmacist/dispensary	Trained	2	2.3 (0.11–8.4)	1	1.0 (0.10–6.1)
	Not trained	86	97.9 (91.6–99.9)	97	99.0 (93.9–100)
Mental health specialists	Trained	2	2.3 (0.12–8.4)	2	2.0 (0.11–7.6)
	Not trained	86	97.7 (91.6–99.9)	96	98.0 (92.4–99.9)

*CI-Confidence interval, HIV-human immunodeficiency virus*

Of the 150 health facilities, 98 (65.3%) had someone who trained on TB diagnosis and care in past two years [Table 3]. Considerable proportion of health facilities had nurses (73.5%) and medical officer (56.3%) who trained on TB diagnosis and treatment in past two years [Table 3]. However, a small proportion (9.2%) of health facilities had nursing assistants who trained on TB diagnosis and treatment in past two years [Table 3]. Of the 98 health facilities which had someone who trained on TB diagnosis and treatment, 32 (32.7%) of health facilities had laboratory personnel, pharmacists 1 (1.0%), and nursing assistants 9 (9.2%) who trained on TB diagnosis and treatment [Table 3].

### Training on non-communicable Diseases and mental Illness management

Of the 150 assessed health facilities, only 43 (28.7%) had someone who trained on non-communicable diseases (NCDs) screening and management in past two years [Table 4]. Of 43 health facilities which had someone who trained on NCDs screening and management, 28 (65.1%) had nurses, and 14 (32.6%) had midwives who trained on NCDs screening and management in past two years [Table 4]. Small proportion (11.6%) of health facilities had nursing assistants and mental health specialist (2.3%) who trained on NCDs management in past two years. Furthermore, only 37 (24.7%) health facilities had someone who trained on mental illness screening and management in past two years [Table 4]. About 60% of health facilities had nurses who trained on mental illness screening and management, while only 13.5% had midwives and 6.3% had medical officers who trained on mental illness screening and management in past two years [Table 4].

**Table 4:** Distribution of training on non-communicable diseases (NCDs) and mental illness management in past two years in health facilities

Variable		NCDs (n = 43)		Mental health (n = 37)	
		Frequency	% (95% CI)	Frequency	% (95% CI)
Nursing	Trained	28	65.1 (50.1–77.6)	22	59.5 (43.5–73.7)
	Not trained	15	10.0 (22.4–49.9)	15	40.5 (26.3–56.5)
Midwifery	Trained	14	32.6 (20.4–47.6)	5	13.5 (5.4–28.5)
	Not trained	29	67.4 (52.5–79.6)	32	86.5 (71.6–94.6)
Nursing assistants	Trained	5	11.6 (4.6–24.9)	3	8.1 (2.1–22.0)
	Not trained	38	88.4 (75.1–95.4)	34	91.9 (78.0–97.9)
Clinical officers	Trained	19	44.2 (30.4–58.9)	13	35.1 (21.8–51.3)
	Not trained	24	55.8 (41.1–69.6)	24	64.9 (48.7–78.2)
Medical officers (n = 16)	Trained	7	43.8 (23.1–66.9)	2	12.5 (2.2–37.3)
	Not trained	9	56.2 (31.2–76.9)	14	87.5 (62.7–97.8)
Mental health specialists	Trained	1	2.3 (0.0–13.2)	3	8.1 (2.1–22.0)
	Not trained	42	97.7 (86.8–100)	34	91.9 (78.0–97.9)

NCDs-non-communicable diseases

### Availability of essential health services

Of the 150 assessed health facilities, 65 (43.6%) had no HIV treatment and follow up service, 49 (32.7%) had no TB treatment and follow up service [Table 5]. About 40% of health facilities had no NCDs screening and management, and BEmoC service [Table 5]. More than 50% of health facilities had no mental illness screening and management service [Table 5]. Immunization services were available in almost all (98.7%) assessed health facilities, and family planning service was provided in 130 (86.7%) health facilities [Table 5].

**Table 5:** Availability of essential health services in drought and food insecurity affected districts

Service availability		Level of health facility, n (%)				
		HC-II (n = 77)	HC-III (n = 56)	HC-IV (n = 9)	Hospital (n = 7)	Total (n = 149)
HIV treatment and follow up	Yes	38 (49.4)	34 (60.7)	7 (77.8)	5 (71.4)	84 (56.4)
	No	39 (50.6)	22 (39.3)	2 (22.2)	2 (28.6)	65 (43.6)
TB treatment and follow up	Yes	49 (63.6)	39 (69.6)	7 (77.8)	6 (85.7)	101(67.8)
	No	28 (36.4)	17 (30.4)	2 (22.2)	1 (14.3)	48 (32.2)
TB/HIV collaboration	Yes	51 (66.2)	35 (62.5)	7 (77.8)	5 (71.4)	98 (65.8)
	No	26 (33.8)	21(37.5)	2 (22.2)	2 (28.6)	51(34.2)
NCDs diagnosis and management	Yes	44 (57.1)	36 (64.3)	7 (77.8)	5 (71.4)	92 (61.7)
	No	33 (42.9)	20 (35.7)	2 (22.2)	2 (28.6)	57 (38.3)
Mental illness screening and management	Yes	33 (47.1)	27 (38.6)	5 (7.1)	5 (7.1)	70 (47.0)
	No	44 (55.7)	29 (36.7)	4 (5.1)	2 (2.5)	79 (53.0)
Family planning service	Yes	70 (90.1)	46 (82.1)	8 (88.9)	6 (85.7)	130 (87.2)
	No	7 (9.1)	10 (17.9)	1 (11.1)	1 (14.3)	19 (12.8)
IMCI	Yes	51(66.2)	46 (82.1)	7 (77.8)	6 (85.7)	110 (73.8)
	No	26 (33.8)	10 (17.9)	2 (22.2)	1(14.3)	39 (26.2)
Immunization	Yes	76 (98.7)	56 (100.0)	9 (100.0)	7 (100.0)	148 (99.3)
	No	1 (1.3)	0 (0.00)	0 (0.00)	0 (0.00)	1 (0.7)
Basic emergency obstetric care	Yes	41 (53.2)	37 (66.1)	5 (55.6)	6 (85.7)	89 (59.7)
	No	36 (46.8)	19 (33.9)	4 (44.4)	1 (14.3)	60 (40.3)

IMCI-integrated management of childhood illness, HIV-human immunodeficiency virus, TB-tuberculosis, NCDs-non-communicable diseases

### Availability of basic laboratory services

Table 6 shows availability of laboratory services across health facilities levels in drought and food insecurity affected districts of north-eastern Uganda. Of the 150 assessed health facilities, 90 (60%) had laboratory services either with laboratory equipment or rapid test. Of the 90 health facilities that had laboratory services, in 79 (87.8%) general microscope service was given, and in 41(45.6%) health facilities malaria rapid test was given. Majority of health facilities (80%), TB smear microscopy test was not provided and in 85 (95.6%) cholera laboratory test with gram stain was not given. Moreover, in few health facilities (13.3%) full blood count, hemoglobin or hematocrit tests, and blood sugar test (5.6%) were provided.

**Table 6:** Availability of laboratory supplies and services in drought and food insecurity affected districts (n = 90)

Service		HC-II (n = 78)	HC-III (n = 56)	HC-IV (n = 9)	Hospital (n = 7)	Total (%)
Any laboratory with laboratory equipment or rapid tests (n = 150)	Yes	45 (50.0)	35 (38.9)	5 (5.6)	5 (5.6)	90 (60.0)
	No	33 (54.2)	21(35.6)	4 (6.8)	2 (3.4)	60 (40.0)
General microscopy	Yes	36 (45.6)	33 (41.8)	5 (6.3)	5 (6.3)	79 (87.8)
	No	9 (81.8)	2 (18.2)	0 (0.0)	0 (0.0)	11(12.2)
Malaria rapid test	Yes	20 (48.8)	16 (39.0)	2 (4.9)	3 (7.3)	41(45.6)
	No	25 (51.0)	19 (38.8)	3 (6.1)	2 (4.1)	49 (54.4)
Malaria microscopy test	Yes	34 (45.3)	31(41.3)	5 (6.7)	5 (6.7)	75 (83.3)
	No	11(73.3)	4 (26.7)	0 (0.0)	0 (0.0)	15 (16.7)
Syphilis rapid test	Yes	40 (47.6)	34 (40.5)	5 (6.0)	5 (6.0)	84 (93.3)
	No	5 (83.3)	1(16.7)	0 (0.0)	0 (0.0)	6 (6.7)
HIV rapid test	Yes	45 (50.0)	35 (39.3)	4 (4.5)	5 (5.6)	89 (98.9)
	No	0 (0.0)	0 (0.0)	1 (100.0)	0 (0.0)	1(1.1)
TB diagnosis test by ZN stain	Yes	6 (33.3)	7(38.9)	3 (16.7)	2 (11.1)	18 (20.0)
	No	39 (54.2)	28 (38.9)	2 (2.8)	3 (4.2)	72 (80.0)
Cholera test (by gram stain)	Yes	1 (25.0)	2 (50.0)	1 (25.0)	0 (0.0)	4 (4.4)
	No	44 (51.2)	33 (38.4)	4 (4.7)	5 (5.8)	86 (95.6)
Urine protein test	Yes	34 (47.9)	28 (39.4)	4 (5.6)	5 (7.0)	71(78.9)
	No	11(57.9)	7 (36.8)	1(5.3)	0 (0.0)	19 (21.1)
Full blood count	Yes	7 (58.3)	2 (16.7)	1 (8.3)	2 (16.7)	12 (13.3)
	No	38 (48.7)	33 (42.3)	4 (5.1)	3 (3.8)	78 (86.7)
Hemoglobin or hematocrit	Yes	7 (58.3)	2 (16.7)	1 (8.3)	2 (16.7)	12 (13.3)
	No	38 (48.7)	33 (42.3)	4 (5.1)	3 (3.8)	78 (86.7)

*ZN-Ziehl-Neelsen, HIV-Human immunodeficiency virus, TB-tuberculosis*

### Availability of tracer medicine

Tracer medicines and stock out status were available in the total of 147 health facilities’ drug stores. Of the observed health facilities, in 74 (50.3%) ampicillin intravenous (IV) and in 70 (47.6%) gentamycin IV were not available [Table 7]. There was no cotrimoxazole in 63 (42.9%) health facilities, and Iron syrup in 129 (87.8%) facilities [Table 7]. Moreover, there was no antiretroviral therapy (ARV) drugs in 74 (50.3) health facilities and anti-TB drugs in 51(34.7%) health facilities [Table 7]. Thirty (20%) of health facilities had stock out at least one tracer medicine on average for 6.3 (± 2.9) days, and considerable proportion (44.9%) of health facilities had no TB preventive therapy (TPT) [Table 7].

**Table 7:** Availability of tracer medicines in health facilities in drought and food insecurity affected districts in Uganda, 2022 (n = 147)

Medicine		Level of health facility, n (%)				
		HC-II (n = 78)	HC-III (n = 53)	HC-IV (n = 9)	Hospital (n = 7)	Total
Amoxicillin	Yes	69 (88.5)	46 (86.8)	9 (100.0)	7 (100.0)	131(89.1)
	No	9 (11.5)	7 (13.2)	0 (0.0)	0 (0.0)	16 (10.9)
Ampicillin IV	Yes	12 (15.4)	46 (86.8)	9 (100.0)	6 (85.7)	73 (49.7)
	No	66 (84.6)	7 (13.2)	0 (0.0)	1()	74 (50.3)
Gentamycin IV	Yes	13 (16.7)	48 (90.6)	9 (100.0)	7 (100)	77 (52.4)
	No	65 (83.3)	5 (9.4)	0 (0.0)	0 (0.0)	70 (47.6)
Vitamin A	Yes	70 (89.7)	47 (88.7)	8 (88.9)	6 (85.7)	131(89.1)
	No	8 (10.3)	6 (11.3)	1 (11.1)	1(14.3)	16 (10.9)
Tetracycline/Chloramphenicol	Yes	58 (74.4)	42 (79.2)	9 (100)	6 (85.7)	115 (78.2)
	No	20 (25.6)	11(20.8)	0 (0.0)	1(14.3)	32 (21.8)
Ciprofloxacin IV	Yes	9 (11.5)	40 (75.5)	6 (66.7)	4 (57.1)	59 (40.1)
	No	69 (88.5)	13 (24.5)	3 (33.3)	3 (42.9)	88 (59.9)
ORS package	Yes	71(91.0)	47 (88.7)	9 (100)	6 (85.7)	133 (90.5)
	No	7 (9.0)	6 (11.3)	0 (0.0)	1 (14.3)	14 (9.5)
Cotrimoxazole	Yes	26 (17.7)	44 (83.0)	9 (100)	5 (71.1)	84 (57.1)
	No	52 (35.4)	9 (17.0)	0 (0.0)	2 (28.6)	63 (42.9)
Zinc	Yes	59 (40.1)	49 (92.5)	9 (100)	6 (85.7)	123 (83.7)
	No	19 (12.9)	4 (7.5)	0 (0.0)	1 (14.3)	24 (16.3)
ACT	Yes	60 (40.8)	49 (92.5)	9 (100)	6 (85.7)	124 (84.4)
	No	18 (12.2)	4 (7.5)	0 (0.0)	1 (14.3)	24 (16.3)
Iron syrup	Yes	9 (6.1)	7 (13.2)	1 (11.1)	1 (14.3)	18 (12.2)
	No	69 (46.9)	46 (92.5)	8 (88.9)	6 (85.7)	129 (87.8)
Iron tablet	Yes	42 (28.6)	33 (62.3)	6 (66.7)	5 (71.4)	86 (58.5)
	No	36 (24.5)	20 (37.7)	3 (33.3)	2 (28.6)	61 (41.5)
Folic acid	Yes	72 (49.0)	48 (90.6)	9 (100)	5 (71.4)	134 (91.2)
	No	6 (4.1)	5 (9.4)	0 (0.0)	2 (28.6)	13 (8.8)
ARV	Yes	13 (8.8)	46 (86.8)	9 (100)	5 (71.4)	73 (49.7)
	No	65 (44.2)	7 (13.2)	0 (0.0)	2 (28.6)	74 (50.3)
Anti-TB drugs	Yes	32 (21.8)	50 (94.3)	9 (100)	5 (71.4)	96 (65.3)
	No	46 (31.3)	3 (5.7)	0 (0.0)	2 (28.6)	51 (34.7)
TPT	Yes	23 (15.6)	44 (83.0)	9 (100)	5 (71.4)	81 (55.1)
	No	55 (37.4)	9 (17.0)	0 (0.0)	2 (28.6)	66 (44.9)

ACT-Artemisinin based combination therapy, ARV-antiretroviral, TB-tuberculosis, TPT-TB preventive therapy, IV-intravenous

### Service quality improvement actions

Of the included 150 health facilities, 91 (60.7%) had quality improvement (QI) committee [Table 8]. Of the total of 91 health facilities which had QI committee, 27 (29.7%) were not holding meeting regularly to discuss quality issues and 66 (72.5%) had no recorded previous QI meeting minutes [Table 8]. Considerable proportion (47.3%) of health facilities had no external quality assessment (EQA) project for essential health services and 89 (59.3%) of health facilities had not conducted quality assessment (QA) in first quarter before the study [Table 8]. Moreover, 113 (75.3%) of health facilities had not documented information on EQA of previous assessment [Table 8].

**Table 8:** Quality improvement status in drought and food insecurity affected districts

Variable		HC-II (n = 78)	HC-III (n = 56)	HC-IV (n = 9)	Hospital (n = 7)	Total (%)
Quality improvement (QI) committee	Yes	35 (38.5)	42 (46.2)	8 (8.8)	6 (6.6)	91 (60.7)
	No	43 (72.4)	14 (24.1)	1 (1.7)	1 (1.7)	59 (39.3)
Continuous professional development assessment	Yes	60 (47.6)	51 (40.5)	9 (7.1)	6 (4.8)	126 (84.0)
	No	18 (75.0)	5 (20.8)	0 (0.0)	1 (4.2)	24 (16.0)
Integrated support supervision (n = 148)	Yes	63 (49.6)	51 (40.2)	7 (5.5)	6 (4.7)	127 (85.8)
	No	15 (71.4)	3 (14.3)	2 (9.5)	1 (4.8)	21 (14.2)
Recent quarter nutrition supportive supervision report (n = 148)	Yes	49 (51.6)	32 (33.7)	8 (8.4)	6 (6.3)	95 (64.2)
	No	29 (54.7)	22 (41.5)	1 (1.9)	1 (1.9)	53 (35.8)
Supportive supervision feedback given	Yes	60 (48.4)	50 (40.3)	7 (5.6)	7 (5.6)	124 (82.7)
	No	18 (69.2)	6 (23.1)	2 (7.7)	0 (0.0)	26 (17.3)
Maternal and child health focal person in QI committee (n = 91)	Yes	32 (37.2)	40 (46.5)	8 (9.3)	6 (7.0)	86 (94.5)
	No	3 (60.0)	2 (40.0)	0 (0.0)	0 (0.0)	5 (5.5)
Data focal person in QI committee	Yes	23 (32.4)	36 (50.7)	7 (9.9)	5 (7.0)	71(78.0)
	No	12 (60.0)	6 (30.0)	1 (5.0)	1 (5.0)	20 (23.0)
TB/HIV focal person in QI committee	Yes	18 (25.4)	39 (54.9)	8 (11.3)	6 (8.5)	71 (78.0)
	No	17 (85.0)	3 (15.0)	0 (0.0)	0 (0.0)	20 (23.0)
Regular QI meeting held	Yes	27 (42.2)	27 (42.2)	5 (7.8)	5 (7.8)	64 (70.3)
	No	8 (29.6)	15 (55.6)	3 (11.1)	1 (3.7)	27 (29.7)
Meeting minute	Yes	11 (44.0)	12 (48.0)	1 (4.0)	1 (4.0)	25 (27.5)
	No	14 (38.9)	15 (41.7)	3 (8.3)	4 (11.1)	66 (72.5)
EQA project for nutrition and essential health services	Yes	16 (33.3)	20 (41.7)	7 (14.6)	5 (10.4)	48 (52.7)
	No	19 (44.2)	22 (51.2)	1 (2.3)	1 (2.3)	43 (47.3)
EQA conducted in facility in past quarter	Yes	22 (36.1)	26 (42.6)	7 (11.5)	6 (9.8)	61 (40.7)
	No	56 (62.5)	30 (34.1)	2 (2.3)	1 (1.1)	89 (59.3)
Previous QA information document	Yes	16 (43.2)	16 (43.2)	2 (5.4)	3 (8.1)	35 (48.6)
	No	12 (34.3)	16 (45.7)	4 (11.4)	3 (8.6)	37 (51.4)
Mentorship of QI	Yes	45 (42.5)	47 (44.3)	8 (7.5)	6 (5.7)	106 (70.7)
	No	32 (74.4)	9 (20.9)	1 (2.3)	1 (2.3)	44 (29.3)

*QI-quality improvement, QA-quality assessment, EQA-external quality assessment*

## Discussion

The current study observed lack of human resource in the health facilities. Majority (91.1%) of health facilities had no mental health specialists. There was also lack of capacity building training for healthcare workers which could have significant impact in provision of quality health services. Considerable proportion of health facilities had no NCDs (38.3%), mental health (47.0%), and BEmOC (40.3%) services. Stock out of essential medicines were observed in 20% of health facilities. There was lack of supportive supervision, documentation and external quality assessment program to improve quality of care across the health facility levels.

Competent and motivated healthcare workers are a critical component of a health system to deliver quality health services. Lack of healthcare workers in the facility is also among the main barriers of access to healthcare services [27]. Furthermore, geographical and socioeconomic disparities in health workforce distribution is a problem across the world [27, 28]. Low and middle income countries (LMICs) are the setup where access to competent healthcare workers is challenging [29]. People living at rural and remote areas in LMICs are more deprived of skilled healthcare workers [30, 31]. Of particular to remote areas, mental healthcare workers shortage is the main problem in LMICs [32], while the burden of mental illness is considerably increasing in these countries [33–35]. This previous evidence is similar with the finding of the current study in which lack of healthcare workers especially mental health specialist in the health facility was observed. Especially, shortage of healthcare workers in lower level health facilities was observed in the current study. This could be due to staff turnover and the national standard of healthcare workers deployment conditions. These deprive rural communities with access to health services from the lower health facilities.

This study shows that a significant proportion of healthcare workers did not get capacity building training on essential health services in past two years across the health facility levels. This could affect the quality of care and the efficiency of the healthcare workers in essential health services delivery. The current study finding is similar with the previous study in which healthcare workers explained lack of training as the main challenge in providing the services [36–38]. Evidence also confirmed the importance of comprehensive ongoing training program for healthcare workers to improve quality of care [39]. It is also known that training given for healthcare workers is important to improve the performance of the healthcare providers and the quality of health services [40]. Thus, in parallel to humanitarian responses on drought and food insecurity emergency, comprehensive training on essential health services is required to improve the performance of the healthcare providers and quality of care to assure continuity of quality of essential health services.

The burden of NCDs is increasing in developing countries [41, 42]. However, unlike developed countries, developing countries lack preparedness in terms of human resources and infrastructure for NCDs diagnosis and management [41, 43, 44]. The current study finding is consistent with the previous results in which considerable proportion of health facilities in low income counties have limited access to NCDs service [44]. In the drought and food insecurity emergency setup, people are suffer from triple burden of diseases such as infectious diseases, malnutrition, and NCDs. This requires an integrated response to manage the health impact of these diseases.

The importance of mental health service has been recently acknowledged and included in sustainable development goals. However, access to mental health service is low across the world [35]. Moreover, access to mental health service is more limited in low income countries [33, 45]. These studies findings are in line with the present study finding in which only 47% of assessed health facilities had mental health service. This could affect the success of drought and food insecurity emergency responses and impact the health and put the population at risk of mental disorder. Therefore, emergency responce agencies and the government should either train the available healthcare workers or deploy the trained mental health specialist to screen and treat mental illness.

In the current study only 40.3% of health facilities had BEmOC service. These findings are similar to the previous review study in which access to BEmOC service was low in Sub-Saharan African countries [46]. Evidence is also indicates that a considerable proportion of mothers are still dying due to lack of BEmOC service in developing countries [47]. Lack of BEmOC service could be more limited in rural and at periphery [48]. These findings are consistent with the present study finding in which health facilities in drought affected rural area had low BEmOC service which could lead to increased child and maternal mortality rate. The problem requires immediate attention to reduce its impact on the health of mothers and the children.

Previous studies have shown that essential medicine stock out is common in health facilities [49–51]. Essential medicines stock out is also significantly affects the treatment process through inducing out of pocket expenditure, treatment non-adherence, service dissatisfaction and low service utilization [50]. These findings are consistent with the present study results in which stock out of essential medicines that last for one week on average were observed.

Quality improvement is vital for better treatment outcomes, health system performance and healthcare worker professional development [52, 53]. However, healthcare facilities are performing below the required levels of quality which leads to deaths of a considerable proportion of patients in LMICs [54, 55]. For instance 15% of deaths occur per year in LMICs is attributable to poor quality of care [54]. Moreover, in several settings quality improvement project in the health facility is not implemented based on recommended standards [56, 57]. These results are similar with the current study finding in which quality improvement activities such as supportive supervision, documentation and external quality assessment program were not well implemented in the assessed health facilities.

The main limitation of this study is lack of evidence on factors that are associated with essential health service delivery from the perspective of patients and community. This could limit the comprehensiveness of the current study findings. Thus, future study should incorporate factors that associated with essential health service delivery and quality improvement actions. Despite the limitation indicated above, the results of this study were less likely to be influenced by those limitations.

## Conclusion

The current study identified that availability of essential health services across health facility levels. However, human resource deployment, particularly mental health specialists, provision of capacity building training for different service areas, improving NCDs, mental health and BEmOC services are required to avail essential health services in health facilities. Moreover, strengthening supply chain management system to minimize stockout of essential medicines, and promoting quality improvement program are vital to improve quality of care and to assure continuity of essential health services under drought and food insecurity emergency affected area.

## Data Availability

The data used in this study is presented in the manuscript, and available from the corresponding author on reasonable request.

## Abbreviations

ACT: Artemisinin based combination therapy
ARV: Antiretroviral
BEmOC: Basic emergency obstetric care
CI: Confidence interval
COVID: 19-corona virus disease 2019
DHOs: District health offices
EQA: External quality assessment
GHoA: Great horn of Africa
HCs: Health centers
HFs: Health facilities
HIV: Human immunodeficiency virus
IMCI: Integrated management of childhood illness
IPC: Integrated food security phase classification
IV: Intravenous
LMICs: Low-and middle-income countries
MoH: Ministry of health
NCDs: Non-communicable diseases
ODK: Open data kit
QA: Quality assessment
QI: Quality improvement
SPSS: Statistical package for social scientists
TB: Tuberculosis
TPT: Preventive therapy
WHO: World Health Organization
ZN: Ziehl-Neelsen
